# Investigating The Antifungal Activity of Atorvastatin Compared with Nystatin on Oral Candidiasis before and during Head and Neck Radiotherapy

**DOI:** 10.31661/gmj.v13iSP1.3717

**Published:** 2024-12-30

**Authors:** Zahra Golestannejad, Faezeh Khozeimeh, Sara Taheri, Soha Feizi, Fatemeh Abbasi, Zahra Saberi, Elham Faghihian, Parvin Dehghan, Faezeh Tadayon, Mahnaz Kheirkhah, Zeinab Nourmohammadi Najafabadi, Zahra Ataie

**Affiliations:** ^1^ Department of Oral and Maxillofacial Medicine, Dental Research Center, Dental Research Institute, School of Dentistry, Isfahan University of Medical Sciences, Isfahan, Iran; ^2^ School of Dentistry, Dental Research Center, Dental Research Institute, Isfahan University of Medical Sciences, Isfahan, Iran; ^3^ Department of Stomatology, Xi’an Jiaotong University, Xi’an, China; ^4^ Department of Oral Medicine, Dental Research Center, Dental Research Institute, Faculty of Dentistry, Isfahan University of Medical Sciences, Isfahan, Iran; ^5^ Department of Mycology and Parasitology, Faculty of Medicine, Isfahan University of Medical Sciences, Isfahan, Iran; ^6^ Department of Dentistry, Dental Students Research Committee, School of Dentistry, Isfahan University of Medical Sciences, Isfahan, Iran; ^7^ Department of Mycology and Parasitology, School of Medicine, Isfahan University of Medical Sciences, Isfahan, Iran; ^8^ Dentistry Department, Isfahan University of Medical Science, Isfahan, Iran; ^9^ Dental Research Center, Dental Research Institute, School of Dentistry, Isfahan University of Medical Sciences, Isfahan, Iran

**Keywords:** Oral Candidiasis, Antifungal Activity, Nystatin, Atorvastatin, Radiotherapy

## Abstract

Background: Radiotherapy plays an imperative role in the control of head and neck malignancies; however, it can damage oral tissues and salivary glands. These damages can alter oral Candida species and lead to the expansion of oral candidiasis resistant to common antifungal mediators, including nystatin. Atorvastatin, a serum cholesterol-lowering drug, has potential antifungal activities by inhibiting the synthesis of ergosterol in the fungal wall and disrupting mitochondrial function. This study aimed to determine and equivalence the antifungal activities of nystatin and atorvastatin on Candida species isolated from the mouths of patients undergoing head and neck radiotherapy before and during radiotherapy. Materials and Methods: This was an in vitro laboratory research conducted on samples isolated from patients experiencing head and neck radiotherapy, before and during radiotherapy. After determining the Candida species using the PCR-RFLP method, the antifungal activity of both nystatin and atorvastatin was evaluated by microdilution method according to CLSI standards, and the minimum inhibitory concentration (MIC) and minimum lethal concentration (MFC) of each drug were measured. Results: According to our findings, atorvastatin had less activity in inhibiting and killing different Candida species compared to nystatin before and during radiotherapy. Before radiotherapy, the MIC and MFC indices for nystatin against Candida albicans (P0.001), tropical (P0.001) and glabrata (P0.001) were significantly lower than these three indices (P-value0.001) for atorvastatin. The results of these indices in the second week of radiotherapy were similar to the results before radiotherapy. The MIC and MFC results for Candida albicans and tropicalis were obtained with a P-value0.001 and for glabrata with a P-value = 0.002. Conclusion: The discoveries of this study indicate that atorvastatin exhibits lower antifungal activity compared to nystatin in treating oral candidiasis among patients receiving head and neck radiotherapy, both before and during the treatment.

## Introduction

One of the effective modalities in controlling head and neck malignancies is radiotherapy. Most of those suffering from these types of malignancies require radiotherapy as primary treatment, after surgery, in combination with chemotherapy, or as palliative treatment. Although radiotherapy has known antitumor activity, ionizing radiation can also damage healthy tissues in the treated area [1][2]. Head and neck radiotherapy often causes damage to oral and facial structures, including the major salivary glands [3] and can lead to complications such as inflammation of the oral mucosa (mucositis), qualitative and quantitative changes in saliva, and dry mouth, about one to two weeks after the initiation of the treatment, which in turn predisposes patient to oral fungal proliferation, colonization, and infections [4][5][6][7].

Dry mouth is one of the symptoms that indicate a decrease in salivary secretion due to radiotherapy of the salivary glands. Atrophy of salivary gland tissue can reduce the quality and especially the quantity of saliva, causing an acidification of salivary pH, a decrease in antimicrobial capacity and a decrease in the cleansing effect of the mouth. All these salivary changes affect oral normal microbial flora, especially Candida species; facilitate the presence of pathogenic agents and increase the risk of oral candidiasis infection [5][8].

Head and neck radiotherapy not only increases the number of fungal species, but also changes their strains [2][9][10]. Candidiasis is the furthermost common clinical infection of the oropharynx in patients undergoing radiotherapy [11]. Studies since 1990 have shown that in addition to Candida albicans, non-albicans species also play a role in the colonization and infection of patients undergoing radiotherapy [6][7][9]. Candida albicans remains the predominant species in oral infections; however, non-albicans species are also emerging as important pathogens, and infections caused by the simultaneous presence of these species are more severe and more resistant to treatment [5][6][12].

Oral candidiasis infections can cause unpleasant complications such as burning sensation in the mouth, pain and dysphagia, which may lead to temporary or complete interruption of radiotherapy, and reduces the antitumor effects of treatment [5]. Moreover, during severe immunosuppression, oral candidiasis can spread to deeper organs and be fatal. Therefore, rapid diagnosis and proper management and treatment of this infection are essential [8]. Among the routine antifungal drugs currently used to control oral candidiasis, topically and systemically; nystatin is a polyene that directly binds to ergosterol in the cell membrane of fungi, leading to the formation of pores in the membrane, disruption of ion balance and cell death. However, the changing epidemiology of fungal infections and the emergence of strains resistant to conventional treatments, including nystatin, have created new challenges in the treatment of these infections [13][14].

Statins, which have been used as cholesterol-lowering agents in recent decades, have multiple effects, including antifungal activity [15]. By inhibiting the HMGCR enzyme, which is involved in the production of ergosterol in fungi, as in humans; these drugs can alter the fluidity and lipid composition of fungal membranes and disrupt cellular metabolism [15][16][17][18].

Considering the inhibitory activity of atorvastatin on fungal growth and the necessity of new drugs in the face of treatment-resistant strains, presen experimental survey objectives to determine the frequency of different oral Candida species in patients with head and neck malignancies before and during radiotherapy and to investigate the sensitivity of these species to nystatin and atorvastatin.

## Materials and Methods

Study Samples

This investigation was conducted as an experimental study (in vitro). The Candida species examined were previously collected and preserved in the Department of Parasitology and Mycology at Isfahan University of Medical Sciences from 33 patients diagnosed with head and neck malignancies at Seyed al-Shohaday Hospital in Isfahan, before and during their radiotherapy treatment. Ethical approval for the research was granted by Isfahan University of Medical Sciences, and informed consent was obtained from all participants before the study commenced. Initially, the cohort comprised 18 women and 15 men, aged between 38 and 74 years, with 21 individuals exhibiting Candida infections prior to the initiation of radiotherapy. During the second week of treatment, 6 patients (four men and two women) succumbed, and 19 out of the remaining 27 patients developed Candida infections. Additionally, some patients were found to be infected with multiple strains of Candida concurrently, such as Candida albicans and Candida tropicalis. The isolated strains were identified and characterized using the restriction fragment length polymorphism PCR (PCR-RFLP) technique. The Candida strains were initially cultured on sub-dextrose agar (SDA) medium and incubated at 35°C for 24 hours to prepare a suspension.Laboratory procedure

To prepare the suspension of the candida, a portion of the Candida species was added to 1 ml of distilled water and the optical absorption of the resulting suspension was measured at a wavelength of 530 nm using a WPA Biowave II spectrophotometer (Biochrom UK). The addition of the species to the suspension continued until its optical absorption reached the range of 0.13-0.08. At this stage, the cell concentration of the resulting suspension was equivalent to 0.5 McFarland. In order to prepare the initial stock of the two antifungal drugs under study, 12.5 mg of nystatin powder (Sigma-Aldrich, Germany) and 12.5 mg of atorvastatin powder (Merck, Germany) were dissolved in 1 ml of methanol and 12.5 mg of atorvastatin powder (Merck, Germany) were dissolved in 1 ml of dimethyl sulfoxide or DMSO (Merck-Germany), respectively; Affording to the Clinical and Laboratory Standards Institute (CLSI) standard; and reserved at laboratory temperature for 30 minutes until the resulting stock was homogenized.

Drug susceptibility testing

To assess the antifungal efficacy of nystatin and atorvastatin, separate evaluations of the minimum inhibitory concentration (MIC) and minimum lethal concentration (MFC) were performed at 24 and 48 hours for various Candida species, including Candida albicans, Candida glabrata, Candida tropicalis, Candida parapsilosis, and Candida krusei. The MIC for both agents was established through a serial dilution technique utilizing 96-well ELISA microplates.For nystatin, ten wells for concentrations of 0.5-128 μg/ml and for atorvastatin, ten wells for concentrations of 8-1024 μg/ml, as well as two positive and negative control wells were prepared. Subsequently, 100 μl of fungal suspension from each species was introduced into each of the ten wells. In the remaining two wells, 100 μl of organism suspension at a density of 1×10³ cells/ml was combined with 100 μl of Roswell Park Memorial Institute (RPMI) medium in the positive control well, while 100 μl of RPMI containing the drug was added to the negative control well, in accordance with the CLSI-M27 guidelines. Following incubation at 35°C for 24 and 48 hours, the turbidity in the wells was assessed, with the first well exhibiting no turbidity identified as the minimum inhibitory concentration (MIC). This process allowed for the determination of MIC24 and MIC48.

To ascertain the minimum lethal concentration (MFC), 100 μl of the suspension from the MIC well and the subsequent wells were transferred to Sabouraud Dextrose Agar (SDA) medium. After performing a sweep culture and incubating for 48 hours at 35°C, the plate that displayed five or fewer colonies of the target species was designated as the MFC.To determine the break point for nystatin, 2µg/ml < MIC48 was considered as drug resistance and 2µg/ml > MIC48 was considered as drug susceptibility. For atorvastatin, since it is not essentially an antifungal drug, a break point is not identified.

Data Analysis

The data were analyzed using SPSS software package, version 22 (SPSS Inc., Chicago, Ill., USA). To investigate the antifungal activity of both nystatin and atorvastatin, three indices MIC24, MIC48 and MFC of each of them were calculated separately on Candida albicans, Candida glabrata and Candida tropicalis species and their median, range and mode were determined. To compare the antifungal activity of these two drugs, the values of the aforementioned indices before and during radiotherapy intervention were analyzed using Mann-Whitney statistical test.

## Results

**Table:** Table1. Median and range of the MIC24, MIC48, and MFC indices of nystatin on strains isolated from the patients before and during radiotherapy intervention

Indices Antifungal activity		Candida albicans		Candida tropicalis		Candida glabrata	
		Before RT	During RT	Before RT	During RT	Before RT	During RT
MIC24	Median Range	1 <0.2-5	0.75 0.1-5	1 0.1-5	1 0.1-5	1 1-1	0.5 0.5-0.5
MIC48	Median Range	1 0.4-5	1.5 0.2-5	2 0.2-5	1.5 2-1	2 2-1	0.5 0.5-0.5
MFC	Median Range	1 0.32-5	1 <0.1-0.5	1 0.1-5	1 0.8-5	1 0.2-5	0.5 0.5-0.5

**Table:** Table2. Median and range of the MIC24, MIC48 and MFC indices of atorvastatin on strains isolated from patients before and during radiotherapy intervention

Indices Antifungal activity		Candida albicans		Candida tropicalis		Candida glabrata	
		Before RT	During RT	Before RT	During RT	Before RT	During RT
MIC24	Median Range	128 256 - 64	128 256 - 128	128 512 - 32	128 512 - 64	128 128 - 64	384 512 - 256
MIC48	Median Range	256 1024 - <128	256 1024 - <256	256 1024 - <64	384 1024 - <256	256 1024 - <256	384 512 - 256
MFC	Median Range	512 1024 - <128	768 1024 - <128	1024 1024 - <128	1024 1024 - <512	1025 1024 - <1024	768 1024 - 512

The Candida samples studied from the fungal collection of the Department of Parasitology and Mycology, Isfahan University of Medical Sciences, included 14 Candida albicans, 5 Candida tropicalis, and 2 Candida glabrata samples before the start of radiotherapy, and 12 Candida albicans, 4 Candida tropicalis, 2 Candida glabrata, 1 Candida parapsilosis, and 1 Candida krusei sample in the second week of radiotherapy.

Tables-1and2 revelaed the median and range of MIC24, MIC48, and MFC indices of the two drugs, nystatin and atorvastatin, against Candida albicans, glabrata, and tropicalis. Antifungal indices were also determined for Parapsilosis and Krusei species, but due to the small number of samples, comparing their frequency distribution was not possible. Also, considering these two species were not isolated from any samples before radiotherapy, comparison of antifungal indices of nystatin with atorvastatin before and during radiotherapy for these two species was not plausible. Based on the findings, MIC24, MIC48, and MFC indices of nystatin against Candida parapsilosis were 0.5, 0.5, and 1 µg/ml, respectively. The MIC24, MIC48, and MFC of atorvastatin were 32, 64, and 128 µg/ml against Candida parapsilosis and 64, 256, and 256 µg/ml against Candida krusei, respectively.

To compare the antifungal activity of nystatin against atorvastatin, the levels of three indices (MIC24, MIC48, and MFC) of these two drugs were separately analyzed by Mann-Whitney once before treatment and once during radiotherapy treatment on each species, and the results are listed in Tables-3and4. Comparison of the antifungal activity of nystatin and atorvastatin showed that the MIC24, MIC48, and MFC indices for nystatin both before and during radiotherapy were significantly lower than atorvastatin (P<0.001). These values were obtained for Candida albicans and tropicalis species with P<0.001 and Candida glabrata species with P = 0.002.

## Discussion

**Table:** Table3. Comparison of antifungal indices (MIC24, MIC48, and MFC) of nystatin against atorvastatin, based on candida species isolated from patients undergoing radiotherapy, before radiotherapy intervention

Indices Antifungal activity		Candida albicans		Candida tropicalis		Candida glabrata	
		Nystatin	Atorvastatin	Nystatin	Atorvastatin	nystatin	atorvastatin
MIC24	Median Range	1 <0.2 - 5	128 256 - 64	1 0.15	128 512 - 32	1 1 - 1	128 128 - 64
P-value		<0.001		<0.001		<0.001	
MIC48	Median Range	1 0.4 - 5	256 1024 - 128	2 0.2 - 5	256 1024 - <128	2 2 - 1	256 11024 - <256
P-value		<0.001		<0.001		<0.001	
MFC	Median Range	1 0.32 - 5	512 1024 - <128	1 0.1 - 5	1024 1024 - <128	1 0.2 - 5	1025 1024 - 1024<
P-value		<0.001		<0.001		<0.001	

**Table:** Table4. Comparison of antifungal indices (MIC24, MIC48, and MFC) of nystatin against atorvastatin, based on candida species isolated from patients undergoing radiotherapy, during radiotherapy intervention

Indices Antifungal activity		Candida albicans		Candida tropicalis		Candida glabrata	
		Nystatin	Atorvastatin	Nystatin	Atorvastatin	nystatin	atorvastatin
MIC24	Median Range	0.75 0.1 - 5	128 256 - 128	1 0.1 - 5	128 512 - 64	0.5 0.5 - 0.5	384 512 - 256
P-value		<0.001		<0.001		0.002
MIC48	Median Range	1.5 0.2 - 5	256 1024 - <256	1.5 2 - 1	384 1024 - <256	0.5 0.5 - 0.5	384 512 - 256
P-value		<0.001		<0.001		0.002	
MFC	Median Range	1 1 - <0.05	768 1024 - <128	1 0.8 - 5	1024 1024 - <512	0.5 0.5 - 0.5	768 1024 - 512
P-value		<0.001		<0.001		0.002	

The findings of the current investigation indicated that prior to and throughout the course of radiotherapy, nystatin exhibited a superior antifungal efficacy compared to atorvastatin against all Candida species obtained from patients receiving head and neck radiotherapy. Specifically, all three antifungal efficacy metrics—MIC24, MIC48, and MFC—were found to be lower for nystatin in comparison to atorvastatin.Atorvastatin’s antifungal effects are primarily mediated through the inhibition of HMG-CoA reductase, which reduces ergosterol synthesis—a critical component of fungal cell membranes. Unlike nystatin, which directly binds to ergosterol and disrupts membrane integrity, atorvastatin’s effect is indirect and less potent at achievable therapeutic concentrations. Recent studies underscore this limitation, noting that while atorvastatin exhibits inhibitory activity against a range of Candida species and azole-resistant strains, its efficacy significantly lags behind nystatin due to lower bioavailability and potential resistance mechanisms, such as alternative sterol synthesis pathways. Furthermore, atorvastatin’s ability to disrupt fungal mitochondria offers an avenue for future exploration, particularly in combination therapies with azoles like fluconazole, which have shown enhanced efficacy against azole-resistant strains. [19][20]

Lima [21] and Nyilasi [22] compared the antifungal activity of nystatin against atorvastatin on Candida species. In the Lima study, which was conducted on fungal strains from the American Cell Bank cultured in mice, the MIC of atorvastatin was lower for Candida albicans and Candida glabrata species and higher for nystatin than current study.

In Candida tropicalis, unlike the previous two species, the MIC of atorvastatin in the Lima study was higher (256 µg/ml) than the present study (128 µg/ml); however, in both studies, the MIC of nystatin activity on Candida tropicalis was almost equivalent.

The study of Nyilasi [22] showed similar results to the present study regarding the antifungal activity of nystatin and atorvastatin on Candida albicans species, so the MIC of atorvastatin and nystatin on Candida albicans species in both studies was 128 µg/ml and 1 µg/ml, respectively. However, the results for Candida glabrata species were different regarding atorvastatin drug and were 32 µg/ml in Nyilasi study and 128 µg/ml in the present study; moreover, the results, the MIC of atorvastatin for Candida glabrata species increased to 328 µg/ml after radiotherapy.

These differences could be due to the difference in the source of the studied species and also the difference in the environment of the isolated Candida species. So in the present study, the change in the quantity and quality of saliva after radiotherapy may lead to a change in the pathogenesis and virulence of the species [23].

The Brilhante study [24] showed that atorvastatin has very different efficacy against different Candida species, with the mean MIC values of 52.6 µg/ml for Candida albicans, 165.34 µg/ml for Candida tropicalis, 755.06 µg/ml for Candida crusei, and 1491.37 µg/ml for Candida parapsilosis.

The results of current study showed that, contrary to the Brilhante’s findings, the MIC for the parapsilosis species was lower than the other studied species and much lower than the results obtained in the Brilhante study (MIC for the parapsilosis species in the present study and in the Brilhante study: 32 µg/ml - 1491.37 µg/ml, respectively). Similar to the parapsilosis species, the Candida crusei species also had a lower MIC in the present study compared to the Brilhante study (MIC for the crusei species in the present study and the Brilhante study: 256 µg/ml and 755 µg/ml, respectively). However, for the Candida albicans and tropicalis, the results of the present study were consistent with the results of his study (MIC for albicans in the present study and the Brilhante study were 128 µg/ml and 6.52 µg/ml, respectively, and MIC for tropicalis in the present study and the Brilhante study were 128 µg/ml and 165 µg/ml, respectively). The difference in the source of the species and the differences in the method used to measure the MIC, including the solvent of atorvastatin in these studies can explain the differences in the results of the current and aforementioned studies. Ting’s systematic study showed that the MIC of statins changes under the influence of factors such as the choice of solvent type, statin form used, methodology and culture medium, and exposure time to statin [25]. In the present study, the solvent type was DMSO, the statin form was pure powder (Pure, Fine, Powdered), the microdilution method, SDA culture medium, and the exposure time was 24 and 48 hours. However, in the Brilhante study, the solvent was sterile distilled water, and the culture medium and statin form were not specified.

Atorvastatin has been widely used as a serum cholesterol-lowering drug since 1990 [26] and has recently been studied as a novel antifungal drug with an inhibitory effect on fungal walls’ biosynthesis [27], so that it may be a suitable alternative for the control of oral candidiasis in cases where the potential for drug resistance is likely. Radiotherapy, by changing the oral environmental conditions including xerostomia, changes in saliva quality, changes in oral pH and mucosal damage (mucositis), leads to changes in the physiological and morphological behavior of Candida species and ultimately changes in the pathogenesis and virulence of yeast [6][23][27][28].

Karbach et al. (2012) shown that decreased salivation following head and neck radiotherapy was associated with the emergence of treatment-resistant Candida strains [29]. Research indicates that alterations in environmental conditions can prompt swift modifications in the gene expression of Candida albicans, facilitating its adaptation to these changes [30]. Paula and Zida determined that 3% of HIV-positive individuals without clinical signs of candidiasis and 4.8% of symptomatic hospitalized patients exhibited resistance to nystatin among various Candida species [31][32]. In contrast to this perspective, the findings of the current study revealed that all strains obtained from patients receiving radiotherapy demonstrated sensitivity to nystatin (MIC < 2 µg/ml) both before and following treatment. This observation aligns with the results reported by Bulacio [33] and Kurnatowski [34].

Furthermore, atorvastatin, even at the maximum concentration tested (1024 µg/ml), still allowed for fungal growth in several instances.Another important hypothesis is that, given the lack of gastrointestinal absorption of nystatin and its only local efficacy, cases of oral candidiasis in people undergoing radiotherapy, who may be immunocompromised and develop candidemia due to factors such as underlying malignancy, previous or concurrent chemotherapy with radiotherapy, or mucositis and subsequent malnutrition [4], require antifungal drugs with systemic and not only local effects. Atorvastatin has gastrointestinal absorption and systemic effects and may be used as a suitable drug in the aforementioned cases where candidemia is likely to occur. The serum level of atorvastatin in Iranians with a daily dose of 40 mg has been reported to be 50 ± 30 ng/ml [35][36][37]. The results showed that the minimum concentration of atorvastatin that can have an inhibitory effect on Candida is 32 µg/ml. Given this concentration is approximately 1000 times the serum level of atorvastatin, this drug cannot be prescribed to control possible candidemia following orofacial candidal infection in individuals undergoing radiotherapy.

Furthermore, the obtained results showed that the antifungal activity of the two drugs (inhibitory effect-killing effect) is not impacted by radiotherapy. While Ramla showed that the production of hydrolytic enzymes including phospholipase and proteinase by the fungus can increases following radiotherapy [6], which itself may change the efficacy of antifungal drugs following radiotherapy. However, since these enzymes are contributed with the fungus ability to colonize at the host tissue and invade the tissue, they do not affect the susceptibility of the species to antifungal drugs in vitro.

While this study indicates that atorvastatin is less effective than nystatin in treating oral candidiasis during radiotherapy, it still holds potential in specific clinical scenarios. For systemic candidiasis, atorvastatin’s gastrointestinal absorption and systemic effects may offer advantages, particularly in immunocompromised patients where localized treatments like nystatin are insufficient.

It is recommended that the antifungal activity of these two drugs compared in vivo in the further studies and to investigate the synergistic effect of atorvastatin when added to the nystatin therapy regimen, rather than comparing the clinical effect of atorvastatin against nystatin.

This study has several limitations. The sample size was relatively small, particularly for certain Candida species such as Candida glabrata, Candida parapsilosis, and Candida krusei, which may limit how widely the results can be applied, and a larger group of participants would provide more reliable

## Conclusion

Overall, the results showed that atorvastatin is not a appropriate alternative to nystatin in patients undergoing radiotherapy.

The antifungal activity of atorvastatin, both before and during radiotherapy, was less than that of nystatin in the treatment of oral candidiasis in patients experiencing head and neck radiotherapy.

## Conflict of Interest

None.
